# Assessment of Digestion and Absorption Properties of 1,3-Dipalmitoyl-2-Oleoyl Glycerol-Rich Lipids Using an In Vitro Gastrointestinal Digestion and Caco-2 Cell-Mediated Coupled Model

**DOI:** 10.3390/molecules29225442

**Published:** 2024-11-18

**Authors:** Hyeon-Jun Chang, A-Young Lee, Jeung-Hee Lee

**Affiliations:** Department of Food and Nutrition, Daegu University, Gyeongsan-si 38453, Republic of Korea; chj931116@naver.com (H.-J.C.); ayounglee@thebreadblue.com (A.-Y.L.)

**Keywords:** Caco-2 cells, in vitro gastrointestinal digestion, in vitro absorption, monolayer tight junction, digestion and absorption property

## Abstract

The digestion and absorption properties of 1,3-dipalmitoyl-2-oleoyl glycerol (POP)-rich lipids was evaluated using in vitro gastrointestinal digestion and a Caco-2 cell-mediated coupled model. Caco-2 cell viability and monolayer integrity were assessed by an MTT assay and transepithelial electrical resistance. The IC50 for bile salts, pancreatin, and free fatty acid (FFA) were 0.22 mM, 0.22 mg/mL, and 1.47 mM, respectively, and no cytotoxicity was observed for bovine serum albumin (0.01–0.20 mM) or triacylglycerol (1.00–10.00 mM). The in vitro-digested POP-rich lipid containing FFA > 2.95 mM caused the disruption of monolayer tight junctions in Caco-2 cells. The major triacylglycerols (TAG) of POP-rich lipids were POP (50.8%), POO (17.8%), POL/OPL/PLO (7.6%), PPO (7.1%), and PLP (6.8%). Following digestion and uptake into Caco-2 cells, the resynthesized TAGs included PPO (20.6%), PPP (15.9%), POO (14.0%), POL/OPL/PLO (12.2%), POP (10.9%), OOO (7.5%), OPO (7.0%), OOL/OLO (6.7%), PLP (3.1%), and PPL (2.2%). The secreted major TAGs were POL/OPL/PLO (50.8%), PPP (11.1%), and OOL/OLO (8.4%), indicating a diverse TAG profile in newly synthesized lipids. This study provides a coupled model for lowering cytotoxicity and maintaining the monolayer in Caco-2 cells, and for evaluating the digestion and absorption properties of functional lipids containing specific fatty acids incorporated into TAG.

## 1. Introduction

The Caco-2 cell line, derived from human colon adenocarcinoma cells, has been widely used as an in vitro absorption model of the intestinal epithelial barrier [1]. The morphological and biochemical features of Caco-2 cells are comparable to those of enterocytes in the small intestine [2]. When grown on a polycarbonate filter, the resulting Caco-2 cell monolayers are useful for evaluating the transport of drug candidates or the absorption of digested lipids and proteins [1,3,4]. In the Transwells^®^, Caco-2 cells can spontaneously differentiate into columnar epithelial cells after reaching confluence, and after 14 days of culture, the microvilli of the cells increase and tight junctions form, and then all structural attributes of intestinal epithelial cells are developed [5]. The tight junctions of cell monolayers can be evaluated by measuring transepithelial electrical resistance (TEER) [3,6], which ranges from 300 to 400 Ω·cm^2^ at 12–25 days post-confluence [7].

Combined models involving in vitro gastrointestinal lipolysis and in vitro absorption by Caco-2 cells have been developed to assess the bioavailability of food lipids [8,9]. In such studies, lipids first underwent lipolysis in an in vitro digestion model (exhibiting an environment similar to human digestion), in which a solution mimicking human pancreatic juice mixed with bile was used, and an intestinal emulsifier (bile salts) and an enzyme complex (pancreatic lipase and pancreatin) were applied. The lipolysis products were then emulsified with an emulsifier (sodium taurocholate, bovine serum albumin, etc.), and after treatment with Caco-2 cell monolayers, in vitro absorption was evaluated. In a coupled study of lipid digestion and absorption, Vors et al. [8] applied rapeseed oil to in vitro gastrointestinal lipolysis, followed by incubation with Caco-2 cell cultures. The conversion of triacylglycerol (TAG) to diacylglycerol (DAG) and monoacylglycerol (MAG) and the release of free fatty acids (FFAs) were measured to assess the lipolysis rate. TAG secretion into the basolateral medium by Caco-2 cells was measured to evaluate absorption. In addition, TEER was measured to assess the integrity of Caco-2 cell monolayer tight junctions [8]. Yan et al. [9] investigated the gastrointestinal digestion fates of lipids using a connected model between pH-stat in vitro digestion and absorption in Caco-2 cells, in addition to the bioavailability of silkworm pupae oil.

Bile salts play a critical role in intestinal lipid digestion by functioning as essential emulsifiers, facilitating the absorption of lipid digestion products [10]. Pancreatic lipase hydrolyzes TAG and DAG, resulting in the formation of 2-MAG and FFAs. The hydrolysis rate of TAG increases as the surface area available for enzymatic activity increases because of the micelles generated by the action of bile salts [11,12]. Pancreatin is an enzyme complex containing lipase, trypsin, amylase, ribonuclease, and proteases secreted by the pancreas [13]. Bile salts, lipases, pancreatin, and emulsifiers should be used at appropriate concentrations based on the absence of toxic effects in in vitro absorption studies in Caco-2 cells, and the FFAs released from the digested lipids should not be harmful to Caco-2 cells. The cytotoxicity of bile salts (sodium taurocholate, sodium deoxycholate, and sodium cholate) and FFAs (e.g., oleic acid) in Caco-2 cells has been previously evaluated [14,15,16,17,18,19,20]. FFAs hydrolyzed from krill and fish oils were reported to be toxic to Caco-2 cells, resulting in decreased cell viability [21].

Previous studies have evaluated the digestion rate of lipids by hydrolyzing TAG-type lipids using in vitro digestion models and by analyzing the released FFA, MAG, DAG, and undigested TAG [22,23,24]. Tailored TAGs with palmitic acid (P) at the *sn*-1,3 position and oleic acid (O) at the *sn*-2 position were synthesized to assess the digestion characteristics by solvent fractionation or enzyme-catalyzed interesterification. Chang and Lee [22] obtained POP-rich lipids from palm stearin by acetone fractionation and assessed their digestibility using multi-step in vitro digestion. Lee et al. [25] extracted POPs from rice bran oil and demonstrated their neuroprotective effects against cerebral ischemia–reperfusion injury. While most studies have been conducted on the digestion or functionality of POPs, research on in vitro absorption or Caco-2 cell cytotoxicity induced by digested POPs remains limited. In the in vitro absorption model, bile salts, pancreatin, emulsifiers, and digested lipid products could induce cytotoxicity in Caco-2 cells. Before applying the in vitro Caco-2 cell-mediated absorption model, it is essential to determine whether specific substances and their concentrations affect the viability and monolayer integrity of Caco-2 cells. If these substances exhibit toxicity, they could reduce Caco-2 cell viability and disrupt the tight junctions of the monolayer, making it impossible to proceed with further absorption experiments.

In the present study, we investigated whether the viability and monolayer integrity of Caco-2 cells were affected by the materials required for the in vitro gastrointestinal digestion of lipids and their hydrolyzed lipid products. Caco-2 cell viability was assessed against bile salts, pancreatin, emulsifiers (sodium taurocholate and bovine serum albumin), and different types of lipids (TAGs, a mixture of acylglycerols, and FFAs) using the 3-(4,5-dimethylthiazol-2-yl)-2,5-diphenyl-2H-tetrazolium bromide (MTT) assay, and the integrity of Caco-2 cell monolayers was assessed by measuring TEER. In addition, the digestion and absorption properties of POP-rich lipids were evaluated by establishing a coupled model of in vitro gastrointestinal digestion and in vitro Caco-2 cell-mediated absorption.

## 2. Results and Discussion

### 2.1. Caco-2 Cell Viability

**Figure 1:**
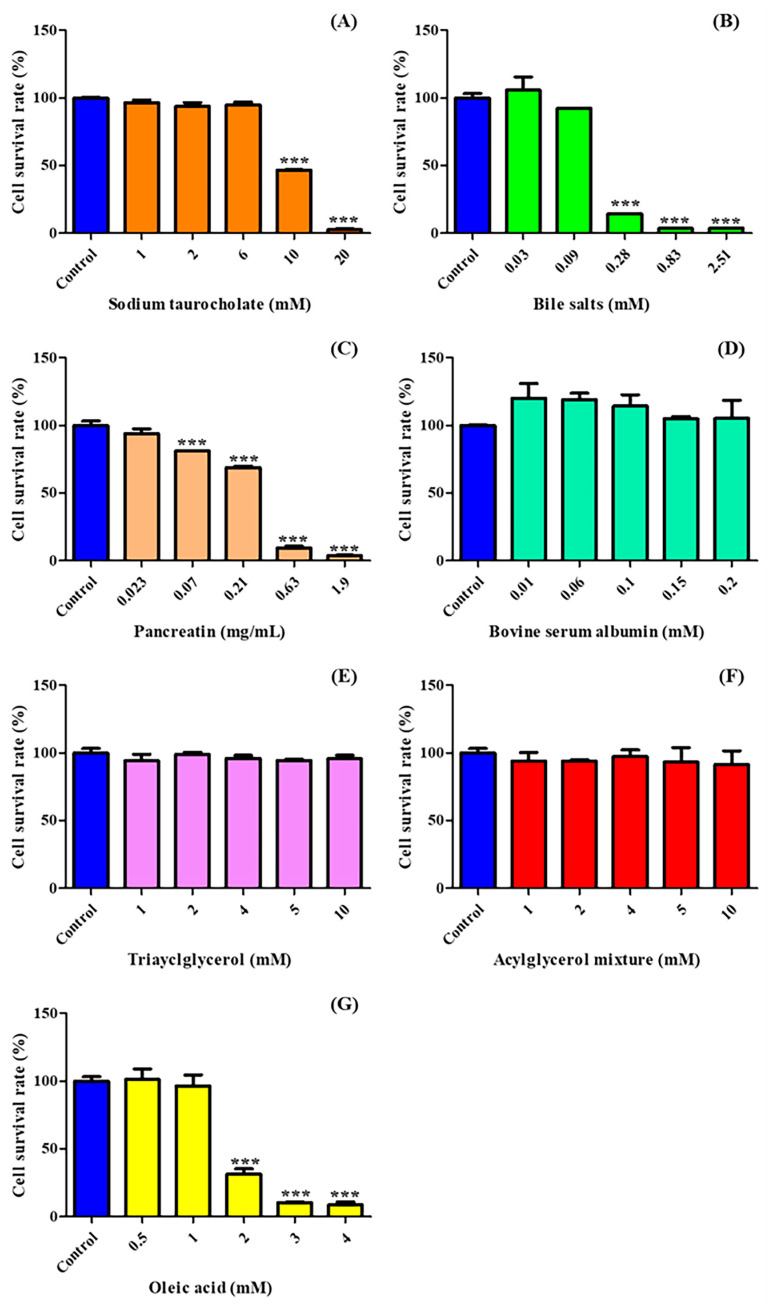
Caco-2 cell viabilities after treatment with sodium taurocholate (**A**), bile salts (**B**), pancreatin (**C**), bovine serum albumin (**D**), triacylglycerol (**E**), acylglycerol mixture (**F**), and oleic acid (**G**). Asterisks indicate a significant difference between the control (untreated group) and each treatment group according to the Dunnett test (*** *p* < 0.001, *n* = 3).

Caco-2 cell viability was measured to determine the cytotoxicity of sodium taurocholate, BSA, bile salts, pancreatin, acylglycerol, and FFAs (oleic acid). The cell viability (%) results are presented as the number of live cells per treatment (Figure 1). Caco-2 cell viability decreased with increasing concentrations of sodium taurocholate, bile salts, pancreatin, and oleic acid. Caco-2 cell viability was 93.81% to 96.59% with 1.00–6.00 mM sodium taurocholate, indicating a lack of cytotoxicity, whereas the viability was considerably reduced to 46.79% at 10 mM and 2.70% at 20 mM (Figure 1A). Caco-2 cell viability values in the presence of 0.03 mM and 0.09 mM bile salts were 105.80% and 92.31%, respectively, indicating no cytotoxicity (Figure 1B). However, the cell viability for the increased concentration of 2.51 mM bile salts was 3.77%, indicating that considerably greater cytotoxicity at higher concentrations adversely affected Caco-2 cells, resulting in a low cell survival rate. The bile salt used in this study was a mixture of 50% sodium cholate and 50% sodium deoxycholate. In the study of Tan et al. [18], sodium deoxycholate at 0.20 mM and 0.40 mM led to Caco-2 cell viabilities of approximately 70% and 60%, respectively, and a concentration of 0.60 mM or higher greatly reduced the viability of Caco-2 cells, resulting in less than 20% cell survival. Yu et al. [26] reported that 10 mM sodium cholate reduced Caco-2 cell viability to a greater extent over time, with the viability reduced by approximately 40% after 24 h.

Bile salts are categorized into three groups based on their conjugation with amino acids and degree of hydroxylation: trihydroxy conjugates (taurocholate and glycocholate), dihydroxy conjugates (glycodeoxycholate, glycochenodeoxycholate, taurochenodeoxycholate, and taurodeoxycholate), and unconjugated forms (cholate, deoxycholate, and chenodeoxycholate) [27]. The IC_50_ values of sodium taurocholate and bile salts were 11.04 mM and 0.22 mM, respectively (Table 1). Meaney and O’Driscoll [14] reported that the IC_50_ of sodium taurocholate for 50% inhibition of mitochondrial enzyme activity was 10.00 mM. Therefore, the present study confirmed that sodium taurocholate is less cytotoxic than the combination of sodium cholate and sodium deoxycholate (bile salts). Bile salts have been reported to induce irreversible mucosal disruption and ciliotoxicity, which limits their clinical use. Bile salts are also cytotoxic to other cell lines. Yang et al. [28] reported that exposing rat brain endothelial 4 cells to sodium deoxycholate resulted in 97% and 100% cytotoxicity at concentrations of 0.50 mM and 1.00 mM, respectively. In a study of the intranasal absorption of gentamicin, Duchateau et al. [29] reported that deoxycholate was highly ciliotoxic at a concentration of 5.00 mM, whereas taurocholate was less ciliotoxic, causing ciliary arrest at 30.00 mM. Among the various bile salts, 1% sodium deoxycholate causes irreversible corneal tissue damage [30].

Pancreatin did not exhibit a cytotoxic effect at 0.02 mg/mL, but the cell viability decreased as the concentration increased so that 0.21 mg/mL resulted in 68.97% viability and 0.63 mg/mL and 1.90 mg/mL resulted in considerable cell damage and reduced cell viability to 9.54% and 3.95%, respectively (Figure 1C). The IC_50_ of pancreatin was 0.22 mg/mL (Table 1), and its cytotoxicity was induced by enzymatic components including trypsin, amylase, lipase, ribonuclease, and protease. The Caco-2 cell viability in the presence of 0.01–0.20 mM BSA ranged from 104.92% to 120.22%, indicating a lack of cytotoxicity. BSA did not exhibit cytotoxicity in Caco-2 cells, even at high concentrations (Figure 1D). The cytotoxicity of BSA has been reported to be low in other cell lines, and the IC_50_ for human dermal fibroblast cells was 2.066 μM, indicating a relatively low level of cytotoxicity compared to other treatment materials [31].

Caco-2 cell viability ranged from 94.20% to 98.90% and 91.39% to 97.33% for TAG (1.00–10.00 mM) and the acylglycerol mixture (1.00–10.00 mM), respectively, with neither exhibiting a cytotoxic effect nor significant variation from the control (*p* > 0.05) (Figure 1E,F). Oleic acid did not exhibit cytotoxicity at concentrations ranging from 0.5 to 1 mM, resulting in 96.55% to 101.28% cell viability. However, as the concentration increased to 4 mM, cell viability was significantly reduced to 8.75% (Figure 1G). The IC_50_ of oleic acid was 1.47 mM (Table 1). Tanaka et al. [19] reported that the viability of Caco-2 cells exposed to long-chain fatty acids (e.g., oleic acid) was not significantly different from that of cells exposed to medium-chain fatty acids (e.g., capric acid, C10:0) or very-long-chain fatty acids (EPA, C20:5; DHA, C22:6) at concentrations up to 1.00 mM for 24 h.

According to Scanferlato et al. [32], treatment of Caco-2 cells with palmitic acid (C16:0) and palmitoleic acid (C16:1, cis9) decreased cell viability and increased cell cytotoxicity in a concentration-dependent manner. In addition, with longer treatment times and higher concentrations, there was an increase in caspase 3/7 and p38 activation (apoptosis markers), as well as in cytosolic phospholipase A2 (cPLA2, involved in cellular signaling pathways related to apoptosis). Notably, the activation levels were higher with palmitic acid (a saturated FA) than with palmitoleic acid (a monounsaturated FA), indicating a relatively lower cell viability with palmitic acid.

### 2.2. Integrity of Caco-2 Cell Monolayer Tight Junctions

**Figure 2:**
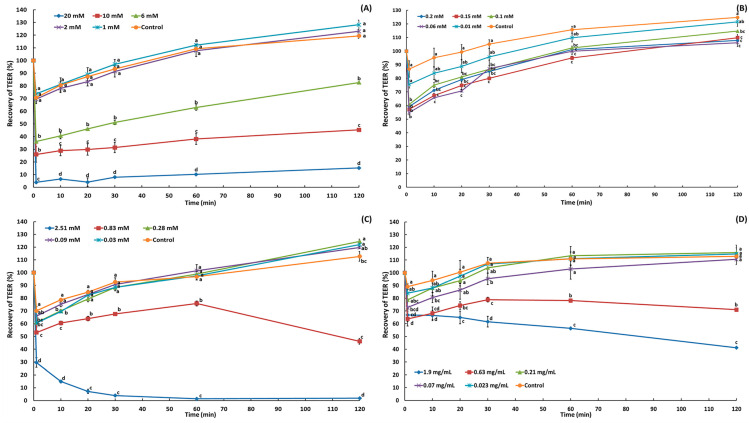
Recovery of transepithelial electrical resistance (TEER) after treatment with sodium taurocholate (**A**), bovine serum albumin (**B**), bile salts (**C**), and pancreatin (**D**) for 2 h. ^a–d^ Means with different letters above the bars at the same time are significantly different at *p* < 0.05 by Duncan’s multiple range test (*n* = 2).

**Figure 3:**
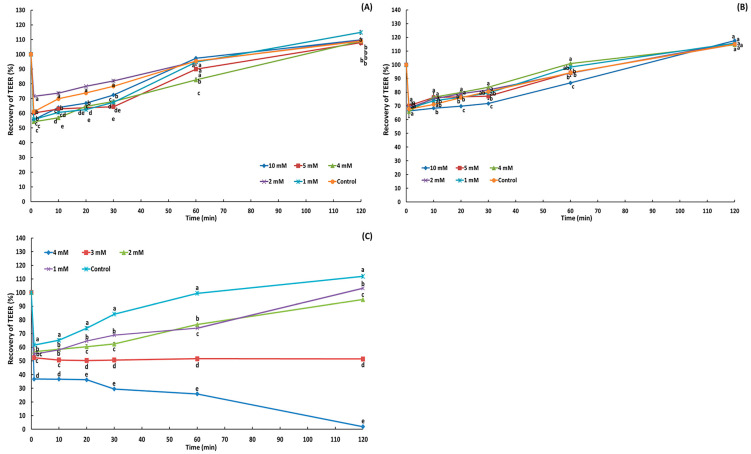
Recovery of transepithelial electrical resistance (TEER) after treatment with triacylglycerol (**A**), the acylglycerol mixture (**B**), and oleic acid (**C**) for 2 h. ^a–e^ Means with different letters above the bars at the same time are significantly different at *p* < 0.05 by Duncan’s multiple range test (*n* = 2).

The TEER value was the main parameter used to assess the integrity and permeability of Caco-2 cell monolayers in the absorption and cytotoxicity assays of the selected materials. The Caco-2 cells used in this study were at passages 34 to 36. The assay to assess monolayer tight junction integrity was conducted with TEER values ranging from 250 to 400 Ω·cm^2^, based on previous studies. According to van Breemen et al. [33], a TEER value of ≥300 Ω·cm^2^ indicated monolayer integrity, while Hubatsch et al. [34] demonstrated that a monolayer formed at a TEER value of 265 Ω·cm^2^. Additionally, studies by Bentz et al. [35] and Matsumoto et al. [36] reported that monolayer integrity was achieved at TEER values of ≥250 Ω·cm^2^. After treatment, the TEER value of Caco-2 cells dropped immediately because the treated materials loosened the tight junctions, decreasing the resistance across the monolayer (Figure 2 and Figure 3). The low TEER values recovered to the initial (before treatment) values when the concentration of the treated materials did not exert any cytotoxicity on the Caco-2 cells, resulting in the restoration of tight junctions [15]. However, some of the decreased TEER values could not be recovered (or even decreased further) when the cells were treated with high concentrations, indicating the cytotoxicity of the materials toward Caco-2 cells (Figure 2 and Figure 3).

The concentration-dependent effects of sodium taurocholate, BSA, bile salts, pancreatin, and acylglycerols on tight junction resistance in Caco-2 cell monolayers were monitored for 2 h, and the results are presented as TEER (%) (Figure 2 and Figure 3). Sodium taurocholate 1.00–2.00 mM resulted in 107.80% to 112.17% recovery in 60 min and 128.22% to 128.22% recovery in 120 min, indicating full recovery of the integrity of tight junctions between Caco-2 cells. In contrast, the TEER (%) values at higher concentrations (6, 10, and 20 mM) were 82.62%, 45.29%, and 15.27%, respectively, after 120 min, confirming that the tight junctions of the Caco-2 cell monolayer were considerably loosened. In a previous study, 10 and 20 mM sodium taurocholate (2 h treatment) led to a decrease in the TEER (%) of the monolayer to 84.90% and 61.80%, respectively, indicating a degree of cytotoxicity toward Caco-2 cells [14]. Mukherjee et al. [15] reported a decrease in the TEER values after 6 h of treatment with 10 and 20 mM sodium taurocholate (900 Ω·cm^2^ decreasing to 700 Ω·cm^2^ and 550 Ω·cm^2^, respectively).

At low concentrations of bile salts (0.03–0.28 mM), a trend of full recovery was observed, as the TEER (%) of the Caco-2 cell monolayer increased to between 119.73% and 124.46% over time. At 0.83 mM, however, the TEER (%) only recovered to 75.85% after 60 min, followed by a decrease to 46.14% after 120 min, and the steep decline continued, reaching 1.85% after 120 min at 2.51 mM, thus confirming the irreversible damage to the Caco-2 cell monolayer. Sakai et al. [16] reported that when cells were treated with sodium deoxycholate at 0.05% and 0.10%, the TEER values decreased to approximately 30% and 10%, respectively, indicating that tight junctions were not retained at these higher concentrations. Liu et al. [17] reported that when cells were treated with 0.20 and 0.30 mM deoxycholic acid for 30 min, TEER values decreased by approximately 50% and 40%, respectively.

At low concentrations of pancreatin (0.02–0.21 mg/mL), the TEER value increased with time, with no significant difference compared to that of the control (*p* < 0.05). But, at 0.63 mg/mL, the TEER (%) increased to 78.86% after 30 min and decreased back to 70.98% after 120 min, while at 1.9 mg/mL, the decline continued, reaching 41.14% after 120 min, confirming considerable destruction of the tight junctions of the Caco-2 cell monolayer (Figure 2D). Keemink and Bergström [37] reported that the concentration (900 USPU/mL) of the pancreatic extract enzyme from pancreatin decreased the TEER value (%) by 50% in Caco-2 cells. After BSA treatment (0.01–0.20 mM), TEER (%) increased with time (Figure 2B). Treatment with 0.01 mM displayed no significant variation from the control (*p* > 0.05), while treatment with 0.06–0.20 mM led to values of 106.19% to 114.71%, indicating full recovery of the integrity and lack of an influence on Caco-2 cell monolayer tight junctions.

The integrity of Caco-2 cell monolayers measured for acylglycerol (Figure 3) showed that TEER (%) increased over time for TAG (1–10 mM) and the acylglycerol mixture (TAG/DAG/MAG, 56.83%/36.06%/7.11%, *w*/*w*), reaching 107.90% to 114.86% and 114.62% to 117.63%, respectively, after 120 min, indicating full recovery. However, for the treatment with oleic acid at 1.00 and 2.00 mM, the TEER values increased over time, reaching between 94.97% and 103.19% recovery after 120 min, whereas the TEER value at 3 mM decreased to 51.42%, and at 4.00 mM, the decline continued, reaching 1.92% recovery, indicating complete destruction of the Caco-2 cell monolayer tight junctions.

Oleic acid resulted in greater monolayer tight junction disruption than TAG or the acylglycerol mixture. The IC_50_ value of oleic acid was 1.47 mM, whereas those of TAG and the mixture could not be calculated because concentrations up to 10.00 mM did not induce Caco-2 cell death. Oleic acid impaired tight junctions between Caco-2 cells in a dose-dependent manner, resulting in a 50% reduction in the TEER value (3 mM) and complete disruption (4 mM) after 2 h of exposure. Similarly to oleic acid, excessive butyrate (C4:0) (>5 mM) significantly reduced the TEER value of Caco-2 cell monolayers, indicating that apoptosis caused by butyrate-induced cytotoxicity led to markedly lower Caco-2 cell viability and significantly disrupted intestinal barrier function [38]. Butyrate induces apoptosis in Caco-2 cells via the intrinsic pathway; as the concentration increases, Bcl-2 and Bcl-xL levels decrease, cytochrome c and Bak levels increase, and caspase-1 and -3 levels increase [39]. The impact of dietary fatty acids with different unsaturation on the integrity of Caco-2 cell monolayers showed that a saturated fatty acid-rich mixture (0.2 mM palmitic acid + 0.1 mM stearic acid + 0.2 mM oleic acid) reduced the TEER value more than a monounsaturated fatty acid-rich mixture (0.35 mM oleic acid + 0.075 mM palmitic acid + 0.075 mM linoleic acid) and a polyunsaturated fatty acid-rich mixture (0.325 mM linoleic acid + 0.1 mM oleic acid + 0.05 mM palmitic acid + 0.025 mM stearic acid) after 4 days of exposure [40].

### 2.3. In Vitro Gastrointestinal Digestion of POP-Rich Lipids

The fatty acids of the POP-rich lipids were composed of palmitic acid (PA) and oleic acid (OA) as the major fatty acids, accounting for 86%, with linoleic acid and stearic acid (SA) in smaller amounts (Table 2). The fatty acids at the *sn*-2 position were oleic, palmitic, linoleic, and stearic acids, while the fatty acids at the *sn*-1,3 position were palmitic (63.25%), oleic (24.98%), linoleic (4.27%), and stearic acids (6.14%). For in vitro gastrointestinal digestion, pancreatic lipase, a lipid digestive enzyme secreted by the mammalian pancreas, is an *sn*-1,3 regiospecific lipase with no stereoselectivity which initially converts TAGs to 1,2(2,3)-*sn*-diacylglycerols and eventually to 2-*sn*-monoacylglycerols [41]. The major FFAs released from the TAG were palmitic (51.82%) and oleic (34.74%) acids, followed by linoleic (6.68%), stearic (5.55%), and myristic (1.21%) acids in smaller amounts. The fatty acids esterified in the 2-MAG were predominantly oleic acid (65.43%), followed by palmitic (16.96%), linoleic (15.80%), and stearic acids (1.81%). Therefore, among the fatty acids bound at the *sn*-1,3 positions of TAG, unsaturated fatty acids (oleic and linoleic acids) underwent hydrolysis more efficiently than saturated fatty acids (palmitic and stearic acids).

The TAGs of the POP-rich lipids were mainly composed of SaMoSa (major, POP), SaMoMo (major, POO), SaMoD/MoSaD/SaDMo (major, POL/OPL/PLO), SaSaMo (major, PPO), and SaDSa (major, PLP), with relative compositions of 50.8%, 17.8%, 7.6%, 7.1%, and 6.8%, respectively, and small amounts of MoMoMo (major, OOO), SaSaSa (major, PPP), and MoMoD/MoDMo (major, OOL/OLO), with relative compositions of 3.3%, 3.2%, and 3.2%, respectively (Table 3). After in vitro gastrointestinal digestion by Method II, the TAG content of the digested POP rich lipid decreased from 1332.06 nM/mg to 280.28 nM/mg, and the relative composition was as follows: SaMoSa (POP, 46.8%) > SaMoMo (POO, 20.8) > SaDSa (PLP, 10.1%) > SaSaMo (PPO, 8.1%) > SaSaSa (PPP, 4.8%) > MoMoD/MoDMo (OOL/OLO, 4.1%) > SaMoD/MoSaD/SaDMo (POL/OPL/PLO, 3.1%) > MoMoMo (OOO, 2.5%). Simultaneously, the concentration of hydrolyzed FFA increased significantly from 0.14% to 48.5%. The major free fatty acids were palmitic (51.82%), oleic (34.74%), linoleic (6.68%), and stearic acids (5.55%), and the fatty acids incorporated at 2-MAG were oleic (65.43%), palmitic (16.96%), linoleic (15.80%), and stearic acid (1.81%) (Table 2). Therefore, the hydrolysis rate of oleic acid (24.98% → 34.74%) from TAG by lipase was higher than that of palmitic acid (63.25% → 51.82%). The digested POP-rich lipids contained palmitic + stearic acids and oleic acid at concentrations of 1075.19 nM and 1000.79 nM/mg, respectively. Lipases from porcine pancreas and *Pseudomonas cepacia* showed higher relative activity for triolein than for tripalmitin, indicating that the hydrolysis rate of oleic acid bound to TAG was higher than that of palmitic acid, which is similar to the results of the present study [42,43].

The in vitro gastrointestinal method used in this study, based on the protocols by Versantvoort [44] and Chang and Lee [45], is more applicable for in vitro gastrointestinal digestion studies involving lipids with small quantities or single components. The INFOGEST method is also widely applied in in vitro simulation of gastrointestinal digestion involving more complex food matrices [46,47]. Both the INFOGEST method and the approach used in this study proceed through the sequential phases of oral, gastric, and intestinal digestion, but they differ in enzyme and chemical selection, concentrations, and sample preparation. The INFOGEST protocol requires a larger sample, using 5 g of food material employing a 1:1 (*w/w*) food-to-simulated oral fluid ratio in the oral phase to form a swallowable bolus. This study, by contrast, used 100 mg of lipid with a 1:12 (*w/w*) ratio. Additionally, pancreatin and pancreatic lipase were applied exclusively in the intestinal phase for the POP-rich lipid in this study; while the INFOGEST protocol includes gastric lipase in the gastric phase, gastric lipase was not employed in this study due to availability constraints. Furthermore, the INFOGEST method generally employs a broader range and higher concentrations of enzymes and reagents than those used in this study.

### 2.4. The Effect of Digested POP-Rich Lipids on the Integrity of Caco-2 Cell Monolayer Tight Junctions

The POP-rich lipids were hydrolyzed using an in vitro multi-step digestion model utilizing methods I and II with different contents of pancreatin and bile salt to investigate whether the digested lipid products would affect the integrity of the tight junctions in the Caco-2 cell monolayer (Table 4). After the cells were treated with digested POP-rich lipids, the FFA concentration in the digested lipids was negatively related to TEER recovery. In the Table 4, the negative value of TEER (%) recovery indicates that the TEER value (Ω) of Caco-2 cells was lower than that of the blank (insert plate with only EMEM).

The FFA content of the POP-rich lipid was 0.14%, and when the lipid was treated with 0.85 mg/mL to 8.46 mg/mL (up to 0.04 mM FFA concentration) in Caco-2 cells, the TEER value remained at 106.64 to 114.77%, indicating that this concentration did not affect the integrity of the cells (Table 4). The digested lipids contained 60.20% and 48.85% FFA with methods I and II, respectively. When treated with the higher concentrations of 2.54 mg/mL and 3.38 mg/mL (from digestion methods I and II, respectively), the TEER (%) value was negative, indicating that the tight junctions of Caco-2 cells did not recover. The FFAs of the digested lipids at 2.54 mg/mL and 3.38 mg/mL were 5.46 mM and 5.90 mM, respectively. Therefore, to evaluate the digestive absorption characteristics of specific lipids using an in vitro Caco-2 cell model, it is necessary to first analyze the concentration of free fatty acids (FFA) released during the in vitro gastrointestinal digestion process.

### 2.5. In Vitro Caco-2 Cell-Mediated Absorption of the Digested POP-Rich Lipids

The digested POP-rich lipids were emulsified with BSA and applied to the Caco-2 cells. The concentration of FFAs in the apical media was 2.95 mM. Over a 48 h incubation period, the TEER recovery (%) of the Caco-2 cell monolayer tight junctions was approximately 100% at 2, 12, and 48 h, confirming no cytotoxicity from the digested POP-rich lipid and the well-maintained integrity of the tight junctions (Table 5).

For the digested POP-rich lipid (MAG and FFA) to be re-esterified into TAGs in Caco-2 cells, the absorbed FFAs (palmitic, oleic, stearic, and linoleic acids) are converted into FA-CoA by long-chain acyl-CoA synthetase, then TAGs are resynthesized via the MAG pathway and the glycerol-3-phosphate (G-3-P) pathway [48]. In the MAG pathway, FA-CoA combines with 2-MAG via the action of MAG acyltransferase (MGAT) to form DAG, which then reacts with FA-CoA by DAG acyltransferase (DGAT) resulting in TAG. In the G-3-P pathway, TAGs are resynthesized from G-3-P and FA-CoA through a series of steps: First, G-3-P undergoes acylation with FA-CoA to form lysophosphatidic acid (lyso-PA) by glycerol-3-phosphate acyltransferase (GPAT). Then, a second acylation step converts lyso-PA into phosphatidic acid (PA). Afterward, PA is dephosphorylated to produce diacylglycerol (DAG). Finally, a third acylation step converts DAG into triacylglycerol (TAG) [48,49]. The primary pathway for TAG resynthesis in Caco-2 cells is via the G-3-P pathway, in which the activity of GPAT is relatively higher than MGAT. The MGAT activity in Caco-2 cells is lower, with less than 7% of activity in the rat jejunum [48,50]. In humans it is known that 70–80% of TAG resynthesis occurs via the MAG pathway [48,49].

In the apical media, 2-MAG and FFAs (mainly palmitic and oleic acids, with small amounts of linoleic and stearic acids) were taken up by Caco-2 cells. The initial concentrations of FFAs gradually decreased and were no longer detectable at 48 h, indicating that most of the FFAs had been absorbed by the Caco-2 cells (Table 5). Each TAG maintained its initial concentration even after 48 h, confirming that it was not taken up by Caco-2 cells and remained in the apical media (Table 5). According to Tsuzuki’s study [51], the absorption rates of 2-monopalmitin and 2-monoolein by Caco-2 cells were similar; however, for FFAs, the absorption rates followed a trend: linoleic acid > oleic acid/palmitic acid > stearic acid, indicating that the more unsaturated the fatty acids, the higher the epithelial transfer rate. Our study showed similar results, with palmitic+stearic acids (P + S) and oleic acid (O) being absorbed at 37.51% and 39.55% after 2 h (Table 5).

Inside the cells, various TAGs were newly synthesized, and these are presented as the sum of each TAG in the basolateral media and inside the Caco-2 cells during each incubation period in Table 3. The original TAG composition of the POP-rich lipid was in the order of SaMoSa (POP) > SaMoMo (POO) > SaMoD/MoSaD/SaDMo (POL/OPL/PLO) > SaSaMo (PPO) > SaDSa (PLP). However, after in vitro digestion and absorption by Caco-2 cells, the resynthesized TAGs for 48 h were in the order of SaSaMo (PPO) > SaSaSa (PPP) > SaMoMo (POO) > SaMoD/MoSaD/SaDMo (POL/OPL/PLO) > SaMoSa (POP) > MoMoMo (OOO) > MoSaMo (OPO) > MoMoD/MoDMo (OOL/OLO) > SaDSa (PLP) > SaSaD (PPL). In the basolateral media, most TAGs (except for PLP and PPL) were secreted by 48 h, with SaMoD/MoSaD/SaDMo (POL/OPL/PLO) being predominant. Among the TAGs, SaSaD (PPL) and MoSaMo (OPO), which were not components of the original POP-rich lipids, were detected only inside the cells and in the basolateral media, indicating that more diverse TAGs were synthesized by the Caco-2 cells.

In the digested POP-rich lipids, the FFA content was higher in P (51.82%) than in O (34.74%), L (6.68%), and S (5.55%), whereas the fatty acid composition of 2-MAG was higher in O (65.43%) than in P (16.96%), L (15.80%), and S (1.81%). However, the content of newly synthesized TAGs with P/S (mainly P) at the *sn*-2 position was higher than that of the TAGs synthesized with O at the *sn*-2 position (Table 3). Therefore, the FA located at 2-MAG and FFA affected the type and amount (degree) of newly synthesized TAGs, showing that TAG synthesis was more efficient when it was positioned at P rather than O at 2-MAG. TAGs with a higher degree of unsaturation were secreted more efficiently. The previous study reported that TAGs esterified with monounsaturated FA and polyunsaturated FA were more easily secreted from the Caco-2 cell than those with saturated SFA [40]. In addition, the slow TAG re-synthesis observed in this study suggests that the digested form of TAG, such as 2-MAG and FFA, results in a longer time required for TAG re-synthesis in Caco-2 cells, which likely use the MAG pathway as a primary route rather than the G-3-P pathway.

## 3. Materials and Methods

### 3.1. Materials

The Caco-2 cell line (colon adenocarcinoma cells) was obtained from the American Type Culture Collection (ATCC^®^, HTB-37, Manassas, VA, USA). Bile salts (B8756, sodium cholate: sodium deoxycholate at 50%:50%), pancreatin from porcine pancreas (P3292, lipase activity: 8 U/mg, amylase activity: 100 U/mg, protease activity: 100 U/mg), pepsin from porcine gastric mucosa (P7000, ≥250 units/mg), lipase from porcine pancreas (L3126, ≥125 units/mg), sodium taurocholate (T4009), bovine serum albumin (A4919, BSA), oleic acid (364525), tripalmitin (PPP), and phosphate-buffered saline were purchased from Sigma-Aldrich Co., Ltd. (St. Louis, MO, USA). Triundecanoin was purchased from NU-CHEK PREP, Inc. (Elysian, MN, USA). α-amylase (12377-1510, ≥8000 U/g) was purchased from Junsei Chemical Co., Ltd. (Tokyo, Japan). Soybean oil (Ottogi Co., Ltd., Anyang, Republic of Korea) was used as the TAG in this experiment. The acylglycerol mixture (TAG, 56.83 *w*%; DAG, 36.06 *w*%; MAG, 7.11 *w*%), synthesized by the lipase-catalyzed glycerolysis of algae oil, was provided by our laboratory [45]. The POP-rich lipids used in this study were obtained from a previous study by the authors and contained mostly TAG (93.40%), with a small amount of DAG (6.33%) and trace amounts of MAG (0.27%) [22]. Serum-free Eagle’s Minimum Essential Medium (EMEM) was obtained from ATCC, and penicillin–streptomycin solution, trypsin, and fetal bovine serum were purchased from HyClone Laboratories, Inc. (Logan, UT, USA). The MTT assay kit was purchased from Elabscience (E-CK-A341, Houston, TX, USA). Transwell^®^ plates (#3460, 12-well, 0.4 μm pore size) and culture dishes were purchased from Corning, Inc. (Corning, NY, USA).

### 3.2. Analysis of Fatty Acid Composition

The sample (50 mg) was saponified and methylated, as described by Shin et al. [23]. The fatty acid composition was analyzed with gas chromatography (Shimazu Corp., Kyoto, Japan) with a flame ionization detector (FID) and SP^TM^-2560 column (100 m × 0.25 mm × 0.2 μm film thickness, Supelco Inc., Bellefonte, PA, USA). Nitrogen (N_2_) was used as the carrier gas with a column flow rate set to 1.0 mL/min, and the injector and detector temperatures were set at 250 °C and 260 °C, respectively. The GC oven was maintained at 100 °C for 5 min, then increased at a rate of 4 °C/min to 240 °C and maintained isothermally for 40 min. Triundecanoin was used as an internal standard, and a Supelco 37 component FAME mixture (Sigma-Aldrich Co., St. Louis, MO, USA) was used as an external standard. The fatty acid composition was expressed as area%.

To determine the positional composition of fatty acids, a sample (10 mg) was mixed with 10 mL of 1 M Tris-HCl buffer (pH 7.6, 10 mL), 0.05% bile salt solution (2.5 mL), 2.2% CaCl_2_ solution (1 mL), and pancreatic lipase (10 mg) in a tube for 1 min. The mixture was incubated at 37 °C for 3 min and vortexed for 30 s (duplicate). Diethyl ether (6 mL) was added, and the mixture was centrifuged (1224× *g* for 5 min). The collected supernatant was concentrated and dried under N_2_ atmosphere. The hydrolyzed sample was dissolved in chloroform and loaded onto a TLC plate. After the 2-monoacylglycerol band on the TLC plate was methylated, the positional fatty acid (FA) composition at *sn*-1,3 was calculated [23].

### 3.3. Caco-2 Cell Culture

Caco-2 cells (passage 34–36) were cultured in complete medium (CM, serum-free EMEM supplemented with 20% fetal bovine serum, 100 units/mL streptomycin, and 100 units/mL penicillin) in an incubator at 37 °C with 5% CO_2_ (MCO175, SANYO Co., Ltd., Osaka, Japan). The medium was changed every two days, and subcultures were performed every two weeks.

### 3.4. MTT Assay for Caco-2 Cell Viability

The cytotoxicity of sodium taurocholate, BSA, bile salts, pancreatin, TAG, the acylglycerol mixture, and oleic acid toward Caco-2 cells was determined using the MTT assay according to a previously published method [38,52,53,54,55], with some modifications. Caco-2 cells were seeded in 96-well plates at a density of 5 × 10^3^ cells/well and cultured in an incubator at 37 °C with 5% CO_2_ for 14 days. The cells in each well were treated with 10.00 μL of the diluted treatment solutions and emulsions and incubated for 2 h. Then, 50.00 μL of MTT solution was added to each well and incubated for an additional 4 h, and the supernatant of each well was discarded. The formazan crystals produced were solubilized by the addition of 150.00 μL of dimethyl sulfoxide, and the optical density (OD) was measured at 570 nm using a microplate reader (Multiskan GO, Thermo Fisher Scientific Ltd., Waltham, MA, USA). Control cells were treated with serum-free EMEM. The Caco-2 cell viability (%) was calculated using the following equation:

$$\mathrm{Cell}\;\mathrm{viability}\;\left(\%\right)=\frac{{\mathrm{OD}}_{570}\;\mathrm{of}\;\mathrm{treated}\;\mathrm{cells}}{{\mathrm{OD}}_{570}\;\mathrm{of}\;\mathrm{control}\;\mathrm{cells}}\times 100$$

The half-maximal inhibitory concentration (IC_50_) of each treated material was calculated based on Caco-2 cell viability (%). For sodium taurocholate and oleic acid, the IC_50_ values were obtained using linear regression, and for bile salts and pancreatin, the IC_50_ values were obtained using log regression.

### 3.5. TEER Measurement of Caco-2 Cell Monolayers

The permeability of the Caco-2 cell monolayers was assessed by measuring TEER (Millipore Corp., Bedford, MA, USA). PET Transwell^®^ inserts (0.4 μm pore) were placed in a 12-well plate. Caco-2 cells were seeded in the apical chambers of the Transwell^®^ inserts at a density of 1 × 10^5^ cells/0.5 mL/well, and 1.50 mL of CM was placed in the basolateral chambers. The cells were cultured in an incubator at 37 °C with 5% CO_2_ for 14 days. The CM in the apical and basolateral chambers was replaced every two days. The experiment was conducted when the TEER value of the Caco-2 cell monolayer ranged between 250 and 400 Ω·cm^2^ (14 days post-seeding) [33]. For the treatment experiments, the CM of the basolateral chambers was replaced with serum-free EMEM, and after removal of the CM from the upper layer, 0.5 mL of each diluted solution of sodium taurocholate, BSA, bile salts, and pancreatin and the diluted emulsions of TAG, an acylglycerol mixture, oleic acid, and digested POP-rich lipids with BSA were placed into each Transwell^®^ insert. The TEER (Ω·cm^2^) was measured after 10, 20, 30, 60, and 120 min of treatment, and the recovery of the TEER value (%) was calculated relative to the TEER value measured for the control without treatment.

$$\mathrm{TEER}\;\left(\%\right)=\frac{\mathrm{TEER}\;\mathrm{value}\;\mathrm{of}\;\mathrm{Caco}-2\;\mathrm{cells}\;\mathrm{after}\;\mathrm{treatment}\;\left(\Omega \cdot {\mathrm{cm}}^{2}\right)}{\mathrm{TEER}\;\mathrm{value}\;\mathrm{of}\;\mathrm{Caco}-2\;\mathrm{cells}\;\mathrm{before}\;\mathrm{treatment}\;\left(\Omega \cdot {\mathrm{cm}}^{2}\right)}\times 100$$

$$\mathrm{TEER}\;\mathrm{value}\;\left({\Omega \;\cdot \;\mathrm{cm}}^{2}\right)=\left\{\mathrm{TEER}\;\mathrm{value}\;\mathrm{of}\;\mathrm{Caco}-2\;\mathrm{cell}\;\left(\Omega \right)-\mathrm{TEER}\;\mathrm{value}\;\mathrm{of}\;\mathrm{blank}\;\left(\Omega \right)\right\}{\;\times \;\mathrm{insert}\;\mathrm{membrane}\;\mathrm{growth}\;\mathrm{area}\;(\mathrm{cm}}^{2})$$

### 3.6. Preparation of the Treatment Materials

For the treatment of Caco-2 cells, sodium taurocholate, BSA, bile salts, and pancreatin were each dissolved in serum-free EMEM, and serially diluted solutions were prepared at the concentrations of 1–10 mM, 0.01–0.2 mM, 0.03–2.51 mM, and 0.02–1.90 mg/mL, respectively. The lipid samples were emulsified using 0.10 mM BSA as an emulsifier. The emulsions of TAG and acylglycerol mixture were prepared at concentrations of 1.00–10.00 mM. The emulsion of oleic acid was prepared at concentrations of 0.50–4.00 mM. Emulsions of POP-rich lipids and digested POP-rich lipids (using Methods I and II) were prepared at 0.85–8.46 mg/mL.

### 3.7. In Vitro Gastrointestinal Digestion of the POP-Rich Lipid

In vitro gastrointestinal digestion of POP-rich lipids was performed by modifying the methods described by Versantvoort [44] and Chang and Lee [45]. Saliva, gastric, duodenal, and bile juices were prepared by mixing each inorganic and organic solution, filling up to 50 mL with distilled water, adjusting to the specified pH, and supplementing them, as presented in Table 6. The methods were modified by adjusting the amount of pancreatin and bile salts added to duodenal and bile juice; 0.90 g and 3.00 g of pancreatin and bile salts, respectively, were applied in Method I, and 0.68 g and 2.30 g of pancreatin and bile salts, respectively, were used in Method II. In vitro gastrointestinal digestion was performed using two methods. POP-rich lipid (100.00 mg) was mixed with saliva juice (1.20 mL) in an Erlenmeyer flask and reacted in a shaking water bath for 5 min at 37 °C and 80 rpm, followed by a 2 h reaction with gastric juice (2.40 mL) and another 2 h reaction with duodenal (2.40 mL) and bile juice (1.20 mL). The digested lipids were extracted with hexane, concentrated, and dried under N_2_ gas. Hexane:ethanol (10 mL; 1:1, *v*/*v*) and 1% phenolphthalein solution (1 mL) were added to the digested POP-rich lipids and titrated with a 0.05 N KOH solution. FFAs (oleic acid equivalents, %) were calculated using the following formula:

$$\mathrm{Acid}\;\mathrm{value}\;\left(\mathrm{mg}\;\mathrm{KOH}/g\right)=\frac{\mathrm{Vol}.\;\mathrm{of}\;\mathrm{KOH}\;\left(\mathrm{mL}\right)\times \mathrm{MW}\;\mathrm{of}\;0.05\;\mathrm{KOH}\;\times \mathrm{Fator}\;\mathrm{of}\;\mathrm{KOH}}{\mathrm{Weight}\;\mathrm{of}\;\mathrm{sample}\;\left(\mathrm{mg}\right)}\times 1000$$

$$\mathrm{Free}\;\mathrm{fatty}\;\mathrm{acid}\;\left(\mathrm{oleic}\;\mathrm{acid}\;\mathrm{equivalent}\;\%\right)=\mathrm{Acid}\;\mathrm{value}\;\times \frac{\mathrm{MW}\;\mathrm{of}\;\mathrm{oleic}\;\mathrm{acid}}{\mathrm{MW}\;\mathrm{of}\;\mathrm{KOH}}\times \frac{100}{1000}$$

### 3.8. In Vitro Caco-2 Cell-Mediated Absorption of the Digested POP-Rich Lipids

Caco-2 cells were seeded at a density of 1 × 10^5^ cells/1 mL/well in the apical chamber of the PET Transwell inserts (6-well plates), and 2 mL of complete medium (CM) was added to the basolateral chamber. The digested POP-rich lipids obtained by Method II of the multi-step in vitro gastrointestinal digestion were emulsified with 0.10 mM BSA. After 14 days of culturing the Caco-2 cells, and once the TEER value reached between 250 and 400 Ω·cm^2^, the emulsion (1.7 mg/mL) was placed into the apical chamber and incubated for 2, 12, or 48 h. During the designated time course, the TEER of the Caco-2 cell monolayer was measured and the apical and basolateral media were collected separately. Because the Caco-2 cells were attached to the insert in the apical chamber, trypsin (1 mL) was added and incubated for an additional one minute, and then removed from the inserts and transferred to a test tube. An internal standard (IS; triundecanoin, 0.1 mg/mL) was added to each test tube (1 mL). Lipids were extracted from the apical media, basolateral media, and Caco-2 cells with Folch’s solution (chloroform:methanol = 2:1, *v*/*v*) by mixing for 1 min. After centrifugation (1224× *g* for 1 min), the lower layer was collected and passed through an anhydrous sodium sulfate column to remove moisture and impurities. The lipid extraction process was repeated twice. The solvent was removed using N_2_, and the extracted lipids were stored at −20 °C for further analysis.

### 3.9. Analysis of TAG Composition in Media and Caco-2 Cells After Incubation with the Digested POP-Rich Lipids

The TAG composition of the lipids extracted from the apical media, basolateral media, and cells was analyzed using silver ion high performance liquid chromatography (Ag-HPLC, Shimadzu Corp., Kyoto, Japan) with an evaporative light scattering detector (ELSD, SEDEX 80LT, Dedere, Alfortvile, France) and a ChromSpher 5 lipid column (250 × 4.6 mm, 5.0 μm, Varian, Middleburg, The Netherlands). The oven and detector temperatures were set at 31 °C and 40 °C, respectively, with an N_2_ gas pressure of 2.5 bar. The extracted lipid was dissolved in hexane at a concentration of 1 mg/mL and injected with a volume of 20 μL. The solvent flow rate was set at 1.5 mL/min, using solvent A (hexane:isopropanol:acetonitrile = 100:0.1:0.1, *v/v/v*) and solvent B (hexane:isopropanol:acetonitrile = 100:1:1, *v/v/v*) as the mobile phases. The solvent gradient was maintained at 100% solvent A from 0 to 5 min, reduced to 80% solvent A at 50 min, decreased to 50% solvent A at 60 min, held for 61 min, increased to 100% solvent A at 62 min, and maintained for 80 min. A tripalmitin solution (standard) was prepared at a concentration of 0.005 mg to 1 mg/mL with the addition of triundecanoin (IS), and the obtained standard curve was used for quantification. With the silver ion HPLC, the TAGs were separated with the number of double bonds and the position (*sn*-1(or 3), *sn*-2) of fatty acids. In the Table 3, TAG was designated with saturated fatty acid (Sa; representing palmitic and stearic acids), monounsaturated fatty acid (Mo; representing oleic acid), and diunsaturated fatty acid (D; representing linoleic acid).

### 3.10. Statistical Analysis

Analysis of variance was performed using Statistical Analysis System 9.2 software (SAS Institute Inc., Cary, NC, USA), and the statistical significance of the results was determined using Duncan’s multiple range test and Student’s *t*-test at *p* < 0.05. GraphPad Prism 5 software (San Diego, CA, USA) was used to perform one-way analysis of variance, and Dunnett’s test was used to determine the statistical significance of Caco-2 cell viability at *p* < 0.001.

## 4. Conclusions

The digestion and absorption properties of the POP-rich lipids were assessed using in vitro gastrointestinal digestion and a Caco-2 cell-mediated coupled model. The IC_50_ values for bile salts, sodium taurocholate, pancreatin, and oleic acid in Caco-2 cells were 0.22 mM, 11.04 mM, 0.22 mg/mL, and 1.47 mM, respectively. No cytotoxic effects or destruction of monolayer tight junctions were observed for BSA, TAG, or the acylglycerol mixture at the concentrations used in this study. The in vitro-digested POP-rich lipid containing FFA higher than 2.95 mM caused the disruption of monolayer tight junctions in Caco-2 cells in the in vitro absorption model. In in vitro gastrointestinal digestion of POP-rich lipids, oleic and linoleic acids are hydrolyzed more extensively than palmitic acid, releasing 2-MAG with highly esterified oleic acid, which is then taken up by Caco-2 cells and synthesized into new TAGs. The POP-rich lipids were initially composed mainly of SaMoSa (POP) and SaMoMo (POO), followed by SaMoD/MoSaD/SaDMo (POL/OPL/PLO), SaSaMo (PPO), and SaDSa (PLP). After in vitro gastrointestinal digestion and uptake into Caco-2 cells, the newly synthesized TAGs composition was more diverse, and their concentrations also changed. Therefore, the composition of FFA and 2-MAG, as well as the TAG synthesis pathway in Caco-2 cells, affected the type and amount (degree) of newly synthesized and secreted TAGs. The findings of this study provide basic data for lowering cytotoxicity and maintaining monolayer tight junctions in Caco-2 cells. Furthermore, this coupled model could be helpful in understanding the digestion and absorption properties of functional lipids containing specific fatty acids incorporated into TAG.

## Figures and Tables

**Table 1:** Inhibition concentrations causing 50% cell death (IC_50_) in Caco-2 cell.

Treatments	IC_50_ ^(1)^
Sodium taurocholate	11.04 mM
Bile salts	0.22 mM
Pancreatin	0.22 mg/mL
Oleic acid	1.47 mM

^(1)^ The IC_50_ value was obtained by constructing a calibration curve based on the results of the MTT assay.

**Table 2:** The fatty acid composition of the POP-rich lipid and digested POP-rich lipid.

Fatty Acids (Area%)	POP-Rich Lipid			Digested POP-Rich Lipid ^(1)^	
	Total	*sn*-1,3	*sn*-2	FFA ^(2)^	2-MAG ^(3)^
C14:0	0.90 ± 0.00 ^(4)^	1.36 ± 0.00	- ^(5)^	1.21 ± 0.00	-
C16:0	46.94 ± 0.04	63.25 ± 0.02	14.32 ± 0.07	51.82 ± 0.38	16.96 ± 0.01
C18:0	4.54 ± 0.01	6.14 ± 0.00	1.33 ± 0.03	5.55 ± 0.99	1.81 ± 0.02
C18:1n-9c	39.28 ± 0.04	24.98 ± 0.00	67.86 ± 0.12	34.74 ± 1.12	65.43 ± 0.08
C18:2n-6c	8.34 ± 0.01	4.27 ± 0.02	16.48 ± 0.02	6.68 ± 0.20	15.80 ± 0.07

^(1)^ In vitro gastrointestinal digestion by Method II. ^(2)^ FFA: free fatty acid. ^(3)^ Fatty acids esterified in 2-MAG (2-monoacylglycerol). ^(4)^ Mean ± SD. (*n* = 2). ^(5)^ Not detected.

**Table 3:** The triacylglycerol composition before and after in vitro gastrointestinal digestion of POP-rich lipids and triacylglycerol composition of re-synthesized TAG by incubation time.

	POP-Rich Lipid (nM/mg of Lipid)		Newly Synthesized TAG ^(1)^ (nM/mg of Lipid)			
	In Vitro Gastrointestinal Digestion		Incubation Time (h)			
	Before	After	0	2	12	48
SaSaSa (PPP)	42.62 ± 4.40 ^(2)^	13.31 ± 0.96	- ^(3)^	9.08 ± 1.25 ^c (4)^	29.75 ± 1.89 ^b^	69.59 ± 1.40 ^a^
SaMoSa (POP)	676.67 ± 24.04	130.99 ± 1.44	-	7.06 ± 0.81 ^c^	18.27 ± 0.11 ^b^	47.62 ± 0.54 ^a^
SaSaMo (PPO)	94.98 ± 4.52	22.62 ± 0.29	-	11.00 ± 0.05 ^c^	39.56 ± 0.87 ^b^	90.41 ± 1.76 ^a^
SaDSa (PLP)	90.35 ± 3.14	28.21 ± 1.15	-	-	7.71 ± 0.21	13.71 ± 0.52 * ^(5)^
SaSaD (PPL)	-	-	-	-	7.07 ± 1.31	9.63 ± 0.41 *
SaMoMo (POO)	239.56 ± 3.88	58.32 ± 0.66	-	9.06 ± 0.35 ^c^	30.82 ± 1.77 ^b^	61.19 ± 0.58 ^a^
MoSaMo (OPO)	-	-	-	7.47 ± 0.18 ^c^	18.85 ± 1.44 ^b^	30.61 ± 1.93 ^a^
SaMoD/MoSaD/SaDMo (POL/OPL/PLO)	101.10 ± 9.03	8.42 ± 0.28	-	-	15.03 ± 0.35	53.67 ± 3.89 *
MoMoMo (OOO)	44.38 ± 3.78	6.86 ± 0.50	-	-	24.75 ± 4.08	32.66 ± 3.41 ^NS (6)^
MoMoD/MoDMo (OOL/OLO)	42.40 ± 1.49	11.56 ± 1.77	-	10.29 ± 1.47 ^c^	21.29 ± 3.38 ^b^	29.26 ± 5.93 ^a^
P + S	-	1075.19 ± 0.92	-	-	-	-
O	-	1000.79 ± 19.27	-	-	-	-
Sum of TAG	1332.06 ± 22.37	280.28 ± 0.94 *	-	53.96 ± 2.48 ^c^	213.09 ± 6.13 ^b^	438.35 ± 7.22 ^a^
Sum of FFA	-	2075.98 ± 18.35	-	-	-	-
FFA released (%)	0.14	48.5				

^(1)^ The sum of the TAG of inside Caco-2 cells and basolateral media. ^(2)^ Sa: saturated fatty acid, Mo: monounsaturated fatty acid, D: diunsaturated fatty acid; SaSaSa (PPP, SSS, PPS, PSP, SSP, SPS; major TAG: PPP), SaMoSa (POP, POS, SOS; major TAG: POP), SaSaMo (PPO, SSO, PSO, SPO; major TAG: PPO), SaDSa (PLP, PLS, SLS; major TAG: PLP), SaSaD/SaMoMo (PPL, SSL, PSL, SPL/POO, SOO; major TAG: PPL/POO), MoSaMo (OPO, OSO; major TAG: OPO), SaDMo/SaMoD (PLO, SLO/POL, SOL; major TAG: PLO/POL), MoMoMo (OOO), MoMoD/MoDMo (OOL, OLO). P: palmtic acid, S: stearic acid, O: oleic acid, L; linoleic acid. ^(3)^ -: Not detected. ^(4) a–c^ Differences between each incubation time of the same lipid was examined for statistical significance with Duncan’s multiple range test (*p* < 0.05). Mean ± SD. (*n* = 2). ^(5)^ * Differences between each incubation time of the same lipid was examined for statistical significance with Student’s *t*-test (*p* < 0.05). ^(6) NS^: not significant.

**Table 4:** Recovery (%) of transepithelial electrical resistance (TEER) after treatment with the POP-rich lipid and digested POP-rich lipid on Caco-2 cells.

Lipid Concentration Treated to Caco-2 Cells (mg/mL)	POP-Rich Lipid		Digested POP-Rich Lipid			
			Method I ^(1)^		Method II ^(2)^	
	TEER Recovery (%)	Free Fatty Acids ^(3)^ Contained (mM)	TEER Recovery (%)	Free Fatty Acids Contained (mM)	TEER Recovery (%)	Free Fatty Acids Contained (mM)
0.85	109.67 ± 0.04 ^bcd (4)^	0.00	98.20 ± 1.65 ^a^	1.82	102.04 ± 1.74 ^a^	1.48
1.69	109.59 ± 0.88 ^bcd^	0.01	85.18 ± 0.65 ^b^	3.64	100.89 ± 0.45 ^a^	2.95
2.54	112.01 ± 1.02 ^ab^	0.01	−9.56 ± 0.13 ^c^	5.46	35.40 ± 0.04 ^b^	4.43
3.38	114.77 ± 1.37 ^a^	0.02	−14.96 ± 1.45 ^ef^	7.28	−9.84 ± 0.92 ^c^	5.90
4.23	109.55 ± 0.67 ^bcd^	0.02	−16.45 ± 0.64 ^f^	9.09	−14.47 ± 0.16 ^e^	7.38
5.08	107.18 ± 0.34 ^cd^	0.03	−12.90 ± 0.17 ^de^	10.91	−11.78 ± 0.06 ^cd^	8.86
5.92	106.64 ± 0.41 ^d^	0.03	−12.10 ± 0.15 ^cd^	12.73	−11.78 ± 1.16 ^de^	10.33
6.77	109.84 ± 0.24 ^bcd^	0.03	−15.14 ± 0.03 ^ef^	14.55	−13.48 ± 0.15 ^de^	11.81
7.61	110.56 ± 2.37 ^bc^	0.04	−17.27 ± 0.87 ^f^	16.37	−15.33 ± 0.22 ^e^	13.28
8.46	108.60 ± 0.44 ^cd^	0.04	−11.57 ± 0.17 ^cd^	18.19	−13.79 ± 0.17 ^de^	14.76

^(1)^ Method I: 0.90 g of pancreatin in duodenal juice and 3.00 g of bile salt in bile juice were used. ^(2)^ Method II: 0.68 g of pancreatin in duodenal juice and 2.30 g of bile salt in bile juice were used. ^(3)^ Calculated as oleic acid equivalents (%). ^(4) a–f^ Means in the same column with different letters are significantly different according to Duncan’s multiple range test at *p* < 0.05. Mean ± SEM (*n* = 2).

**Table 5:** The TAG composition in apical media, basolateral media, and Caco-2 cells during in vitro Caco-2 cell-mediated absorption.

TAG (nM/mg of Lipid)	Apical Media				Basolateral Media ^(1)^				Inside Caco-2 Cells ^(2)^			
	Incubation Time (h)				Incubation Time (h)				Incubation Time (h)			
	0	2	12	48	0	2	12	48	0	2	12	48
SaSaSa (PPP) ^(3)^	9.78 ± 0.71 ^b (4)^	10.85 ± 0.52 ^b^	11.37 ± 1.30 ^b^	14.32 ± 0.28 ^a^	- ^(5)^	-	1.90 ± 0.13	6.03 ± 0.08	-	9.08 ± 1.25 ^c^	27.85 ± 1.77 ^b^	63.56 ± 1.48 ^a^
SaMoSa (POP)	96.25 ± 1.06 ^ab^	99.49 ± 1.25 ^a^	98.34 ± 0.23 ^a^	88.62 ± 6.03 ^b^	-	-	-	2.93 ± 0.01	-	7.06 ± 0.81 ^c^	18.27 ± 0.11 ^b^	44.69 ± 0.54 ^a^
SaSaMo (PPO)	16.62 ± 0.22 ^ab^	16.79 ± 1.13 ^ab^	16.27 ± 1.12 ^b^	18.65 ± 0.12 ^a^	-	-	-	3.08 ± 0.07	-	11.00 ± 0.05 ^c^	39.56 ± 0.87 ^b^	87.33 ± 1.82 ^a^
SaDSa (PLP)	20.73 ± 0.84 ^a^	19.76 ± 1.58 ^ab^	19.44 ± 1.22 ^ab^	17.45 ± 0.03 ^c^	-	-	-	-	-	-	7.71 ± 0.21	13.71 ± 0.52 * ^(6)^
SaSaD (PPL)	-	-	-	-	-	-	-	-	-	-	7.07 ± 1.31	9.63 ± 0.41 *
SaMoMo (POO)	42.85 ± 0.49	43.69 ± 1.83 ^NS (7)^	43.38 ± 2.89	41.21 ± 0.34	-	-	-	3.12 ± 0.04	-	9.06 ± 0.35 ^c^	30.82 ± 1.77 ^b^	58.07 ± 0.62 ^a^
MoSaMo (OPO)	-	-	-	-	-	-	-	3.16 ± 0.14	-	7.47 ± 0.18 ^c^	18.85 ± 1.44 ^b^	27.45 ± 1.79 ^a^
SaMoD/MoSaD/SaDMo (POL/OPL/PLO)	6.18 ± 0.21 ^c^	16.68 ± 1.58 ^b^	24.50 ± 4.64 ^a^	17.23 ± 0.13 ^b^	-	-	-	27.60 ± 0.28	-	-	15.03 ± 0.35	26.06 ± 3.61 *
MoMoMo (OOO)	5.04 ± 0.37 ^b^	11.44 ± 1.79 ^a^	10.89 ± 0.50 ^a^	11.84 ± 0.08 ^a^	-	-	-	3.81 ± 0.06	-	-	24.75 ± 4.08	28.85 ± 3.47 ^NS^
MoMoD/MoDMo (OOL/OLO)	8.49 ± 1.30	16.57 ± 2.39 ^NS^	13.71 ± 4.46	14.58 ± 2.34	-	-	-	4.57 ± 0.03	-	10.29 ± 1.47 ^b^	21.29 ± 3.38 ^ab^	24.69 ± 5.89 ^a^
P + S	790.03 ± 0.67 ^a^	671.89 ± 7.47 ^b^	390.09 ± 22.54 ^c^	-	-	-	-	-	-	-	-	-
O	735.36 ± 14.16 ^a^	605.00 ± 11.56 ^b^	369.80 ± 11.59 ^c^	-	-	-	-	-	-	-	-	-
Total	1731.34 ± 14.17 ^a^	1512.17 ± 5.87 ^b^	997.78 ± 17.76 ^c^	223.89 ± 4.45 ^d^	-	-	1.90 ± 0.13	54.31 ± 0.61 *	-	53.96 ± 2.48 ^c^	211.19 ± 6.01 ^b^	384.05 ± 6.62 ^a^
Sum of TAG	205.95 ± 0.69 ^b^	235.28 ± 1.78 ^a^	237.89 ± 16.36 ^a^	223.89 ± 4.45 ^ab^	-	-	1.90 ± 0.13	54.31 ± 0.61 *		53.96 ± 2.48 ^c^	211.19 ± 6.01 ^b^	384.05 ± 6.62 ^a^
Sum of FFA	1525.40 ± 13.49 ^a^	1276.89 ± 4.08 ^b^	759.89 ± 34.13 ^c^	-	-	-	-	-	-	-	-	-
Recovery of TEER (%)	100.00 ± 0.00 ^NS^	101.38 ± 0.02	101.66 ± 0.02	101.94 ± 0.02	-	-	-	-	-	-	-	-

^(1)^ TAG is secreted from Caco-2 cells into the basolateral media. ^(2)^ TAG is resynthesized in Caco-2 cells and remains within the cells. ^(3)^ Sa: saturated fatty acid, Mo: monounsaturated fatty acid, D: diunsaturated fatty acid; SaSaSa (PPP, SSS, PPS, PSP, SSP, SPS; major TAG: PPP), SaMoSa (POP, POS, SOS; major TAG: POP), SaSaMo (PPO, SSO, PSO, SPO; major TAG: PPO), SaDSa (PLP, PLS, SLS; major TAG: PLP), SaSaD/SaMoMo (PPL, SSL, PSL, SPL/POO, SOO; major TAG: PPL/POO), MoSaMo (OPO, OSO; major TAG: OPO), SaDMo/SaMoD (PLO, SLO/POL, SOL; major TAG: PLO/POL), MoMoMo (OOO); MoMoD/MoDMo (OOL, OLO), P: palmitic acid, S: stearic acid, O: oleic acid, L; linoleic acid. ^(4) a–d^ Differences between each incubation time of the same lipid were examined for statistical significance with Duncan’s multiple range test (*p* < 0.05). Mean ± SD. (*n* = 2) ^(5)^ -: Not detected. ^(6)^ * Differences between each incubation time of the same lipid was examined for statistical significance with Student’s *t*-test (*p* < 0.05). ^(7) NS^: not significant.

**Table 6:** The composition of synthetic juices for in vitro gastrointestinal digestion model ^(1)^.

	Saliva Juice	Gastric Juice	Duodenal Juice	Bile Juice
Inorganic solution	1 mL KCl (89.6 g/L)	1.57 mL NaCl (175.3 g/L)	4 mL NaCl (175.3 g/L)	3 mL NaCl (175.3 g/L)
	1 mL KSCN (20 g/L)	0.3 mL NaH_2_PO_4_ (88.8 g/L)	4 mL NaHCO_3_ (84.7 g/L)	6.83 mL NaHCO_3_ (84.7 g/L)
	1 mL NaH_2_PO_4_ (88.8 g/L)	0.92 mL KCl (89.6 g/L)	1 mL KH_2_PO_4_ (8 g/L)	0.42 mL KCl (89.6 g/L)
	0.17 mL NaCl (175.3 g/L)	1.8 mL CaCl_2_·2H_2_O (22.2 g/L)	0.63 mL KCl (89.6 g/L)	1 mL CaCl_2_·2H_2_O (22.2 g/L)
	2 mL NaHCO_3_ (84.7 g/L)	1 mL NH_4_Cl (30.6 g/L)	1 mL MgCl_2_ (5 g/L)	15 μL HCl (440.3 g/L)
		0.65 mL HCl (440.3 g/L)	0.9 mL CaCl_2_·2H_2_O (22.2 g/L)	
			18 μL HCl (440.3 g/L)	
Organic solution		1 mL Glucose (65 g/L)		
	0.8 mL Urea (25 g/L)	1 mL Glucuronic acid (2 g/L)	0.4 mL Urea (25 g/L)	1 mL Urea (25 g/L)
		0.34 mL Urea (25 g/L)		
		1 mL Glucosamine (33 g/L)		
Supplementation to the solution	α-amylase 29 mg	BSA 0.1 g	BSA 0.1 g	BSA 0.18 g
	Uric acid 1.5 mg	Pepsin 0.25 g	Pancreatin 0.9 g (I) ^(2)^ or 0.68 g (II) ^(3)^	Bile salt 3 g (I) or 2.3g (II)
	Mucin 2.5 mg	Mucin 0.3 g	Lipase 0.15 g	
pH	6.8 ± 0.2 ^(4)^	1.3 ± 0.02	8.1 ± 0.2	8.2 ± 0.2

^(1)^ Versantvoort et al. [44]; Chang and Lee [45]. ^(2)^ Method I: 0.9 g of pancreatin in duodenal juice and 3.0 g of bile salt in bile juice were used. ^(3)^ Method II: 0.68 g of pancreatin in duodenal juice and 2.3 g of bile salt in bile juice were used. ^(4)^ Mean ± SD.

## Data Availability

The original contributions presented in the study are included in the article, further inquiries can be directed to the corresponding author.
